# The longitudinal course of non-suicidal self-injury and deliberate self-harm: a systematic review of the literature

**DOI:** 10.1186/s40479-014-0024-3

**Published:** 2015-01-30

**Authors:** Paul L Plener, Teresa S Schumacher, Lara M Munz, Rebecca C Groschwitz

**Affiliations:** Department of Child and Adolescent Psychiatry and Psychotherapy, University of Ulm, Steinhoevelstr. 5, 89075 Ulm, Germany

**Keywords:** Non-suicidal self-injury, Deliberate self-harm, Predictors, Longitudinal study, Systematic review

## Abstract

Non-suicidal self-injury (NSSI) has been proposed as diagnostic entity and was added to the section 3 of the DSM 5. Nevertheless, little is known about the long-term course of this disorder and many studies have pointed to the fact that NSSI seems to be volatile over time. We aimed to assemble studies providing longitudinal data about NSSI and furthermore included studies using the definition of deliberate self-harm (DSH) to broaden the epidemiological picture. Using a systematic search strategy, we were able to retrieve 32 studies reporting longitudinal data about NSSI and DSH. We furthermore aimed to describe predictors for the occurrence of NSSI and DSH that were identified in these longitudinal studies. Taken together, there is evidence for an increase in rates of NSSI and DSH in adolescence with a decline in young adulthood. With regards to predictors, rates of depressive symptoms and female gender were often reported as predictor for both NSSI and DSH.

## Introduction

Non-suicidal self-injury (NSSI) has been proposed as a new diagnostic entity in the section 3 (conditions for further study) of the fifth edition of the Diagnostic and Statistical Manual (DSM 5) [1]. The introduction of this new category has been discussed extensively [2–4], with strong arguments both for the implementation (such as i.e. avoiding to falsely label adolescent self-injurers as having borderline personality disorder, and addressing a topic with a high prevalence rate) and for the opposite (i.e. incorrectly calling a behavior “non-suicidal” which is a clear risk factor for suicide attempts). There is still an ongoing debate about how to correctly define self-harming behaviors, with part of the scientific community using the term Deliberate Self Harm (DSH) to describe any self-directed harmful behaviors (indirect or direct), regardless of their suicidal intent [5,6]. In contrast, NSSI defines only directly harmful behaviors without suicidal intent [1].

It remains undisputed that NSSI is a very prevalent phenomenon among adolescents, with lifetime prevalence rates of at least one self-injuring event around 18% in community samples worldwide [7,8]. First studies using the proposed section 3 DSM 5 criteria reported rates between 4% and 7% for adolescent community samples and around 50% for child and adolescent psychiatric samples (for review: see [9]). A recent review also found prevalence rates of NSSI and DSH in adolescents to be comparable [7]. A recent comparison of 12 European countries (using the definition of “direct self-injurious behavior”, which seems close to both a NSSI and a DSH definition), reported a mean prevalence rate of 27.6% in adolescents reaching from 17.1% in Hungary to 38.7% in France [10]. There are only very few studies assessing rates of NSSI in adult community samples. Klonsky reported a 5.9% lifetime prevalence rate of NSSI using a random digit dialing sample from the US [11]. This inconsistency of high lifetime prevalence rates in adolescence and rather low lifetime prevalence rates in adults [8] requires further exploration. One explanation might be a re-attribution in adulthood, considering adolescent NSSI as being “nothing important to report”, which would lead to underestimation in studies about lifetime NSSI rates in adults. Furthermore, there might be a memory bias of adults not remembering how frequently they had engaged in NSSI in adolescence. Alternatively, it might be possible that NSSI has increased in recent years, however, no indication for a rise of prevalence rates was found in the two systematic reviews of the literature [7,8]. Given that both NSSI and DSH are highly prevalent in community sample and emerge in adolescence, research about predictors of these behaviors are highly relevant as they could inform preventive interventions. As predictors can be best identified through longitudinal research, studies focusing on a longitudinal assessment have the potential to inform researchers and policy makers.

Given this questions, it seems worthwhile to pay a closer look to the longitudinal development of NSSI and DSH. We therefore performed a systematic literature review to include every study that assessed NSSI and DSH longitudinally. Aims of this review were to (1) assess the stability of prevalence rates of NSSI and DSH over time. Further, (2) to compare 12-month incidence rates of NSSI and DSH in adolescents and adults and (3) to identify predictors for NSSI and DSH that have been reported constantly in longitudinal research.

## Review

**Figure 1:**
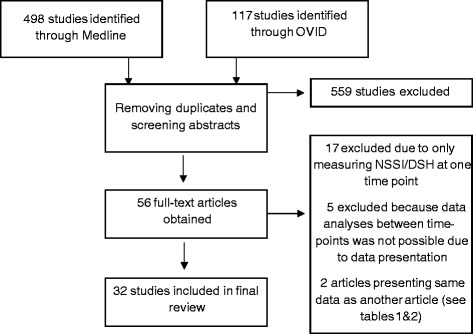
**Flow-chart of study selection.**

We performed a systematic review of the literature using Medline and OVID. As a search strategy we used the terms “NSSI”, “DSH”, “self-harm”, “self-injury”, “deliberate self-harm”, “nonsuicidal self-injury” in conjunction with “longitudinal” or “course”. Only studies written in English or German providing longitudinal data about NSSI or DSH and published before the 1^st^ of August 2014 were included. Only studies measuring NSSI/DSH at - at least - two consecutive points in the same individuals were included. We excluded studies focusing on self-harm in populations with pervasive developmental disorders or mental retardation or studies providing longitudinal data about predictors of NSSI or DSH but just measuring NSSI/DSH at one point in time (see Figure 1). This led to the inclusion of 43 studies (see Tables 1 and 2).

**Table 1:** **Longitudinal studies of NSSI from community and clinical samples (n = 22)**

**Authors**	**Country**	**population**	**Age at baseline**	**N**	**Follow-up period**	**Outcome**
Community samples						
You et al., 2012 [16]	China	Community sample	Mean age: 14.63 (SD: 1.25)	2435	6 months	Baseline: 24.9% (12 month prevalence)
						T1: 13.9% (6 month prevalence)
						10.7% of sample: NSSI at both time points
Franklin et al., 2014 [17]	USA	Community sample	Mean age: 24.37 (SD: 8.28)	49	6 months	Baseline: 100%, (cutting: sum: 248, mean: 5.06, SD: 7.44) Follow-up: 20 reported no cutting during the follow-up (but at baseline); cutting: sum:164, mean: 3.42 (SD: 6.08)
Wan et al., 2014 [18]	China	Community sample	Mean age: 16.1 (SD: 2.8), age range: 12–24 years	13923	9 months	Baseline NSSI: 17.0% (12 month prevalence)
						3 months follow-up: 10.5% (3 month prevalence)
						6 months follow-up: 7.8% (3 month prevalence)
						9 months follow-up: 8.8% (3 month prevalence)
Hasking et al., 2013 [26]; Tatnell et al., 2014 [36]	Australia	Community sample	Mean age: 13.89 (SD: 0.97), age range: 12–18 years	1973	11.7 months	Baseline: 8.3% (lifetime prevalence)
						T1: 11.9%
						3.8% initiated NSSI
Modén et al., 2013 [15]	Sweden	Register study of all adults in Scania	Adults	936449	12 months	Incidence rate: 91/100 000 (male), 128/100 000 (female)
						19.3% of males with recent NSSI have injured themselves in the three years before, as well as 23.9% of females
Hamza & Willoughby, 2014 [19]	Canada	University sample	Mean age: 19.15	666: 466 with past or recent NSSI + 200 controls without NSSI from a larger sample of 1153	12 month	Baseline: 38% (lifetime prevalence)
						T1: 2% (incident NSSI)
						Beginners: New NSSI at T1: 5.72% of participants with NSSI
						Recovered: lifetime NSSI but no NSSI since one year before baseline: 41,31%
						Relapsers: lifetime NSSI, no NSSI one year prior to baseline but NSSI prior to T1: 9.96%
						Desisters: NSSI in 12 month prior to baseline but not in 12 month prior to T1: 28.39%
						Persisters: NSSI 12 months prior to baseline and T1: 14.62%
You et al., 2014 [20]	China	Community sample	Mean age: 14.63 (SD: 1.25)	3600	12 months	Baseline: 10.3% (6 month prevalence)
						T1: (6 months follow-up): 12.7%
						T2 (12 months follow-up): 9.2%
Martin et al., 2014 [37]	Australia	Community sample	Mean age: 14.87 (SD: 0.95)	1896	12 months	Baseline: 6%
						T1: 12 months after baseline: 3.7% (incident NSSI)
Prinstein et al., 2010 (Study 1) [12]	USA	Community sample adolescents	8th grade	377	12 months	Baseline: 7.4% (12 month prevalence)
						After one year: 3.2%
Glenn & Klonsky, 2011 [21]	USA	College sample screened for NSSI	Mean age: 18.96 (SD: 1.57)	Baseline: 81 12 month follow-up: 51	12 months	Baseline: 100% (lifetime prevalence)
						52% (6-month prevalence)
						12 month follow up: 62.7% NSSI (12 month prevalence)
Whitlock et al., 2012 [38]	USA	College sample	Mean age: 20.3 (SD: 4)	1466	24 months	Baseline: 13.7% (lifetime prevalence)
						New NSSI at year 1: 5.2%
						New NSSI year 2: 0.8%
						Cumulative prevalence: 19.7%
Marshall et al., 2013 [27]	Sweden	Community sample	Mean age: 13.21 (SD: 0.57)	506	24 months	Baseline: 0.20 (6 months: mean of Deliberate Self Harm Inventory item scores)
						T1: 0.24
						T2: 0.25
Barrocas et al., 2014 [22]	China	Community sample	Mean age: 16.02 (SD: 0.61)	617	24 months (assessment every 3 months)	T1 (3 months after baseline): 23.8%
						T2: 17.6%
						T3: 17.2%
						T4: 11.4%
						T5: 13.8%
						T6: 12.2%
						T7: 11.5%
						T8: 11.1% (all 3 months prevalence)
Voon et al., 2014 [28]	Australia	Community sample	Mean age: 13.9 (SD:. 0.99)	3143	24 months	Baseline: 8.1%
						T1: 24 months after baseline: 10,1% (lifetime prevalence)
Hankin & Abela, 2011 [29]	USA	Community sample	*M* age = 12.63, *SD* = 1.25	97 at both waves	30 months	Baseline: 8% (12 month prevalence)
						Follow up: 18% newly initiated: 14%
						Continuation: 50% (n = 4)
Baetens et al., 2014 [30]	Belgium	Community sample	Mean age: 12 years	533 (all time points)	30 months	Baseline: 5.15% lifetime prevalence
						T1: 12 months after baseline: 2.78% (12 month prevalence)
						T2: 30 months after baseline: 5.31% (12 month prevalence)
						Cumulative: 10.70% (lifetime prevalence)
Clinical samples or clinical studies						
Rosenbaum Asarnow et al., 2011 [39]	USA	Participants of depression treatment study x	Mean age: 14.2 (SD: 1.2)	327	6 months	Baseline: 23.9% NSSI alone, 14% NSSI and suicidal attempt
						T1: 11% incidence rate
Wilkinson et al., 2011 [40]	UK	Participants of depression treatment study	Mean age: 14.2 (SD: 1.2)	163	7 months	Baseline: 36% (1 month prevalence)
						T1: 37% (during follow-up)
Guerry & Prinstein, 2010 [23]	USA	Child and adolescent psychiatric inpatients	Mean age: 13.51 (SD: 0,75), age range: 12–15 years	143	18 months	Baseline: 67,9% (12 month prevalence)
						T1: 3 month: 32.7% (last 3 month)
						T2: 6 month: 29.0%
						T3: 9 months: 34.0%
						T4: 15 months: 22.8%
						T5: 18 months: 28.4%
Prinstein et al., 2010 (Study 2) [12]	USA	Child and adolescent psychiatric inpatients	Mean age: 13.51 (SD: 0,75), age range: 12–15 years	140	18 months	Baseline 1.14
						T1: 9 months: 1.11, T2: 18 months: 1.10 (mean score of NSSI behaviors: 12 month prevalence)
McGlashan et al., 2005 [24]	USA	Patients with personality disorders	Adults, age range: 18-45	474 (201 with Borderline Personality disorder)	24 months	Baseline: 60%
						T1: 24 months: 30%, remission in 46%
Tuisku et al., 2014 [25]	Finland	Adolescent outpatients	Mean age: 16.5	139	96 months	Baseline: 32.4%
						T1: 12 months after baseline: 21.7% (12 months prevalence)
						T2: (96 months after baseline): 16.1%

T1: first assessment after baseline.

T2-Tx: consecutive assessments.

N is provided for the last wave of the respective studies to describe participants being included in the longitudinal design.

Studies are sorted by follow-up time-frame.

**Table 2:** **Longitudinal studies of DSH from community and clinical samples (n = 9)**

**Study**	**Country**	**Population**	**Age at baseline**	**N**	**Follow-up period**	**Outcomes**
Community samples						
O´Connor et al., 2009 [31]	Scotland	Community sample	Mean age: 15.2 (SD: 0.72), age range: 15–16 years	500	6 months	baseline: 9.5% (12 month prevalence)
						T1: 6.2% (6 month prevalence)
						2.6% for the first time
						3.6% repeaters
Lundh et al., 2011 [41]	Sweden	Community sample	Grade 7 and 8	879 at both waves	12 months	baseline: 45.1% (female) and 37.9% (male) (6 month prevalence)
Bjärehed et al., 2012 [42]						
						incidence rate: 10.4% (female), 8% (male)
Larsson & Sund, 2008 [33]	USA	Community sample adolescents	Mean age: 13.7 (SD: 0.58), age range: 12–15 years	2360	12 months	baseline: 4.2%
						T1: 12.7% (12 months)
						incidence rate: 2.4%
Stallard et al., 2013 [43]	UK	Community sample	12-16 years	3955	12 months	baseline: 9.6% (6 month prevalence)
						T1: 10.9% (6 month prevalence)
						cumulative: 15% DSH during study period
						55.1% continued DSH after one year
Wichstrom, 2009 [44]	Norway	Community sample adolescents	Mean age: 16.5 years (SD: 1.9)	2924	60 months	baseline: 2.4% (lifetime prevalence)
						T1: 2.2% (during follow-up period)
						9.9% of baseline DSH continued
Rossow & Norström, 2014 [32]	Norway	Community sample	Mean age: 16.5, age range: 14-21	2647	60 months	baseline: 3.2% (12 month prevalence)
						T1: 1.6%
						stable DSH at both points: 0.30%, decrease in DSH: 2.9%
						new DSH. 1.3%
Moran et al., 2012 [13]	Australia	Community sample	Mean age: 15.9 (SD 0.49)	1652 (responding to questions of DSH at least once in adolescence and once in adulthood)	174 months	first assessment of DSH at wave three (baseline of DSH): 5.1% (12 month prevalence)
						at wave 9: 0.5% (6 month prevalence)
						any self harm during adolescence: 8.3%, any self-harm during young adulthood: 2.6%
						only cutting/burning: 4.6% in adolescent phase
						1.2% in young adult phase
						new DSH in young adulthood: 1.6% remission in young adulthood: 7.4%, continuation in young adulthood: 0.8%
Clinical samples/samples from hospitals						
Hawton et al., 2012 [45]	UK	Individuals presenting with self harm to hospital	0-18 years	5205	72 months	Repetition of DSH: 27.3%
		17.1% of self harm epidsodes: self-injury				
Sinclair et al., 2010 [46]	UK	Clinical sample: self harm patients: 94% self-poisoning, 4% self-injury, 2% both self-poisoning and self-injury	Median age: 28.4 years	143	74 months	Further self-harm in 57.4%
Wedig et al., 2012 [14]	USA	Clinical sample: Patients with Borderline personality disorder	Mean age: 26.9 years	231	192 months (16 years, Tx every 2 years)	Baseline: 90.3%
						T1: 50.9%
						T2: 35.3%
						T3: 28.4%
						T4: 22.4%
						T5: 17.7%
						T6: 23.0%
						T7: 18.5%
						T8: 14.3%

T1: first assessment after baseline.

N is provided for the last wave of the respective studies to describe participants being included in the longitudinal design.

## Results

Of the 32 studies selected, 22 (69%) represented studies on NSSI, whereas ten studies (31%) used a definition of DSH (see Tables 1 and 2). Combining both, 24 (75%) of the studies presented data from community samples and nine studies (25%) reported data from clinical samples or from clinical studies (including the study by Prinstein et al. [12], presenting data both from a community and a clinical sample; for details see Tables 1 and 2). Twenty-five (78%) of all studies reported on participants who were in their adolescence during baseline.

On average, duration of follow-up was 19.53 months (SD: 18.76) in studies concerning NSSI and 67.4 months (SD: 66.7) in studies concerning DSH. Although the huge difference was driven by the studies by Moran et al., [13], and Wedig et al., [14] who provided a follow-up for around 15 and 16 years respectively concerning DSH, exclusion of these outliers still yielded a result of a longer follow-up period in DSH studies (M = 38.5 months, SD = 30.4). Number of participants encompassed N = 969,197 in studies on NSSI (mean number of participants: N = 44,054; SD = 199,341). As this number was mainly due to the large number of participants from a registry study [15], calculations after exclusion of this study showed smaller numbers (N = 32,748; mean number of participants: N = 1,559; SD = 3,025). Overall, 20,496 individuals participated in longitudinal studies of DSH (mean number: N = 2,049, SD = 1,688).

### Developmental course of NSSI and DSH

**Figure 2:**
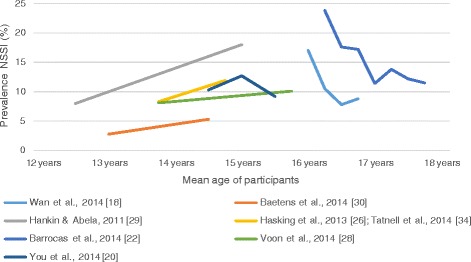
**Studies on prevalence of NSSI in adolescent community samples.** Only studies giving information about mean age of participants, and which used the same prevalence measures for each time-point, were included. For individual prevalence time-frames (i.e. 3-months, 6 months, etc.) of each study see Table 1.

It was possible to retrieve data about decline or increase of rates from 17 studies on NSSI and five studies on DSH. There was a decrease in rates of NSSI during the follow-up in 12 ([12]: both study populations; [16–25]) and an increase in five ([26–30]) of these 17 studies on NSSI. The course of NSSI throughout adolescence is shown in Figure 2. However, only seven studies provided data on community samples of adolescents measuring the same time-frame of prevalence (i.e. 6-months prevalence) of NSSI at all time-points.

Interestingly, five out of the six studies showing an increase of rates of NSSI, were performed in younger adolescents. A decrease in NSSI was mainly found in older adolescents and adults (see Figure 2 and Table 1).

An incidence rate of NSSI within a 12 month time frame was provided in five studies of NSSI in community samples of adolescents and young adults (mean incidence rate: 4.32%, SD: 1.08). In studies using a DSH definition, a decline was described in four ([13,14,31,32], and an increase in one [33] of the five eligible studies (see Table 2).

### Predictors of NSSI and DSH

Analyzing the predictors of NSSI and DSH, only longitudinal predictors (existing at or before baseline) for NSSI during or at follow-up were included. A similar pattern could be found for NSSI and DSH. For NSSI, the predictor cited most often was previous NSSI, followed by depression, female gender, suicidality and psychological distress (see Table 3).

**Table 3:** **Predictors and protective factors described in longitudinal studies using a NSSI definition**

**Domain**	**Predictor**	**Number of studies**	
Personal		Predictor for NSSI during follow-up, existing before baseline	Predictor for NSSI during follow-up, measured at baseline
Gender	Female gender	11	
NSSI	(Previous) NSSI	6	6
	Lifetime NSSI methods	1	
	NSSI thoughts		1
	Low implicit and explicit aversion to NSSI stimuli		1
	Forecasting future NSSI		1
Suicidality	History of suicide attempt	3	
	Suicidal ideation		3
Psychiatric symptoms	Depressive symptoms		7
	Conduct disorder/problems		3
	Anxiety		2
	BPD features (e.g. emotional reactivity, unstable relationship, unstable sense of self-image)		2
	Drug and alcohol use impairment		2
	Neurologic or psychiatric disease		1
	Persistent psychotic experiences		1
Psychological impairment	Psychological distress		5
	Hopelessness		2
	Emotional problems		1
	Problem behaviors		1
	Lower self-esteem		1
	Greater internalizing problems		1
	Behavioral impulsivity		1
	Negative emotions		1
	Rumination		1
	Attachment anxiety		1
Life events	More stressful or negative life events		2
	Early sexual debut		1
	Physical and sexual abuse		1
Other	Negative attributional style		3
	Negative cognitive style		1
	Excessive reassurance seeking		1
	Being single (male)		1
	Non-heterosexual sexual interest		1
	Younger age		1
Family	Onset of parental depression		1
	Lower perceived family support		1
	Problems with parents		1
Social	Friends’ engagement in NSSI		2
	Social adaption problems/		1
	Problems with peers		1
	Lack of social support		1
	Relationship problems		1
	Negative interactions		
Protective	Higher self esteem		2
	Social support		1
	Cognitive reappraisal		1
	Parental care		1

Number of studies: Number of studies describing the specified predictor.

Only predictors for NSSI at follow-up were included.

For DSH, higher scores of depression were described as predictor in the majority of studies, followed by female gender, lower self-esteem and alcohol/ drug use. Past behavior (previous DSH) as predictor of future DSH was described in two studies. Overall, numerous predictors overlapped in studies of NSSI and DSH.

## Conclusion

Performing a systematic review of the literature, we were able to retrieve 32 studies, which assessed either NSSI or DSH longitudinally. Overall, both NSSI and DSH showed high volatility between assessment points with some studies reporting an increase, and some reporting a decrease of self-harming behaviors over time. Even within studies, there were high rates of discontinuation vs. new initiation of self-harming behaviors. A pattern emerged in studies of NSSI with studies being performed in young adolescents showing an upward trend in rates of NSSI, whereas studies in older adolescents or young adults showed a decrease in rates.

This could point to a natural course of NSSI with an increase in young adolescence and a decrease in late adolescence/young adulthood. The only study, which covered the whole time range from adolescence to adulthood, so far, is that of Moran et al. [13]. Although the study focused on self-harm, thus also including suicidal behavior, the authors described a decrease in self cutting/burning behavior from adolescence to adulthood. Since this study is the most long-lasting and one of the largest studies in this field of research, it seems possible to suggest that both NSSI and DSH peak in adolescence at around 15 to 17 years and then remit in young to middle adulthood. Describing NSSI as a behavior that seems to change quickly, it makes sense to restrict possible diagnostic criteria of NSSI disorder to a short time span. As proposed in section three of the DSM 5 [1], there should be repeated incidents of NSSI within a year to define the behavior. NSSI that has remitted for longer than a year or is not repetitive in its nature should not be classified as a disorder.

With regards to a developmental course, it would be interesting to follow up a broad range of risk behaviors over time. NSSI has been described as strong risk factor for later suicidality repeatedly (for review see [34]), with suicide attempts also increasing in adolescence for the first time [35]. Future studies therefore need to shed light on possible changes in behavior, i.e. NSSI possibly diminishing over time but being substituted by suicidal behavior or other risk seeking behavior such as illicit drug use.

Looking into predictors reported from longitudinal research, there seems to be an overlap between studies focusing on NSSI and those focusing on DSH. Namely, among the predictors found in several studies on DSH and NSSI, past self-harming behavior seems to be one of the strongest predictors for future behaviors. In addition, depressive symptomatology as well as female gender were reported in multiple studies. Knowledge about predictors could aid the development of preventive interventions, which could focus i.e. on the detection of depressive symptoms and need to be tailored for gender.

In addition to prevention programs, early interventions programs need to be established, focusing on those already injuring themselves and trying to stop the self-harming behavior which in itself is a predictor of future behavior. Several reviews have also identified social and family factors contributing to NSSI and DSH, suggesting preventive interventions should also address this topic (such as by integrating strategies against bullying).

However, due to the vast heterogeneity of the studies included in this review, general comments about the longitudinal course and predictors of NSSI/DSH are not easy to come up with. First, this is due to the different definitions of self-injurious behaviors used across studies. Further, assessment tools ranged from one question (i.e. “have you ever intentionally harmed yourself”) to extensive interviews. Also, a number of studies did not assess self-injury homogeneously at all time-points (i.e. lifetime-NSSI at baseline and 3-months prevalence at follow-up), which made interpretation difficult at times. Moreover, follow-up periods were very heterogeneous, ranging from several months to 16 years. Therefore, a standardized definition of self-injurious behaviors and an establishment of evaluated tools for measuring NSSI seems to be well needed.

Limitations: This review presents a wide range of studies with different aims, therefore description of predictors and according to the numbers of studies in which the predictors were described is in no way meant as weighing predictors against each other. As some studies have looked at special aspects of NSSI or DSH, the range of predictors that were examined was restricted. Nevertheless, there seems to be a pattern of certain predictors that were described repeatedly and consistently throughout studies, which seems to be an interesting finding. Further, only studies in English or German language were included. This was due to language skills of the authors and limited resources, which did not allow for translation of articles in all relevant languages.
